# Acceptability, Feasibility, and Perceived Effectiveness of Video-Based Patient Records for Supporting Care Delivery to Older Adults With Frailty: Nonrandomized Mixed Methods Pilot Study

**DOI:** 10.2196/77318

**Published:** 2026-01-06

**Authors:** Phoebe Averill, Rachael Lear, Ricky Odedra, Susannah Long, Alex Taylor, Pi-Jung Charville, Jessica Fernandes, Uzoamaka Ekeogu, Jessica Leombruno, Sophia Ellis, Erik Mayer

**Affiliations:** 1 NIHR North West London Patient Safety Research Collaboration, Institute of Global Health Innovation, Imperial College London, St Mary’s Hospital, London, W2 1NY, United Kingdom; 2 Better Health & Care Hub, King’s College London, London, United Kingdom; 3 Robin Hood Lane Health Centre, Sutton, United Kingdom; 4 iCARE Secure Data Environment, NIHR Imperial Biomedical Research Centre, Imperial College Healthcare NHS Trust, London, United Kingdom; 5 Imperial College Healthcare NHS Trust, London, United Kingdom

**Keywords:** frailty, video recording, digital health, patient safety, quality of care

## Abstract

**Background:**

Frailty constitutes a growing challenge for health and social care systems around the world. In England, 35% of adults aged 65 years and older live with frailty, with international estimates indicating that almost half of all hospital inpatients within the same age group are frail. This population often experiences multimorbidity and frequent care transitions. Written documentation and verbal handovers may lack the precision and nuance required to understand an older adult’s presentation and support needs. Video recordings of individual patients, capturing aspects of their functional abilities and condition, may help to enhance multidisciplinary team communication and care continuity, yet little is known about their use in the care of older inpatients with frailty.

**Objective:**

We aimed to evaluate the acceptability, feasibility of implementation, and perceived effectiveness of video-based patient records (the Isla Health Digital Pathway Platform) for supporting the assessment and care of older inpatients with frailty within the acute hospital setting.

**Methods:**

A nonrandomized mixed methods pilot study was conducted within 3 acute medicine wards for older adults. The video-based patient records intervention, permitting videos to be embedded securely within the electronic patient record, was implemented over a 3-month period alongside usual care. Patient enrollment and retention figures; qualitative interviews with patients, carers, and clinical staff; and video capture and view metrics were used to address the study objectives. The Theoretical Framework of Acceptability of Healthcare Interventions was applied to the framework analysis of interview data, capturing concepts such as intervention ethicality, burden, and coherence. Patient and public involvement and engagement informed each research stage.

**Results:**

Twenty-nine patients were enrolled (56.9%); 1 patient withdrew before receiving the intervention. Modal reasons given by patients for nonparticipation included not wanting to take part in research (n=8) or feeling too unwell (n=2). Staff identified multiple opportunities for capturing patient videos, including documentation of mobility assessments or seizures. The intervention was considered acceptable on the grounds that safeguards were always in place, including secure data storage and upholding of patient dignity. Implementation barriers and facilitators were identified; factors such as difficulties in capturing videos within busy ward environments and scheduling issues were voiced by participants. Video view metrics and data from interviews collectively suggested low rates of engagement with videos by clinical staff once captured. Potential intervention impacts included perceived enhancements to clinical assessment and person-centered care.

**Conclusions:**

Our findings suggest that the intervention is largely acceptable to patients, carers, and clinical staff. Conclusions as to intervention feasibility were mixed, with limited engagement with videos suggesting further work is required to promote sufficient uptake among staff. Finally, this research presents promising patient, carer, and clinical opinion as to the potential effectiveness of video-based patient records for improving aspects of patient care.

## Introduction

### Background

Frailty can be understood as impairment in a person’s ability to recover following an injury or illness [1]. It is commonly characterized by exhaustion upon low energy expenditure, diminished muscle strength, and unplanned weight loss [2], and represents a pertinent challenge for health and social care systems globally in the context of population aging [3]. Over one-third (35%) of adults in England aged ≥65 years are thought to live with frailty [4], with a synthesis of international literature indicating a pooled prevalence rate of 47% for frailty among hospital inpatients in the same age group [5].

Older adults with frailty typically have complex care needs, experience multimorbidity, and require input from several care providers spanning multiple, fragmented health and social care organizations [6,7]. Evidence suggests that such patients are particularly vulnerable to experiencing shortfalls in safety and quality of care, owing to poor care continuity [8,9]. This can contribute to avoidable deconditioning in older adults [10], who may struggle to return to their usual activities and routines upon discharge from the hospital to community-based care.

The World Health Organization acknowledges the inherent risks to patient safety at transitions of care, where treatment delays, duplication of tests, and increased rates of adverse events are among identified challenges [11]. Frequent transitions between health and social care settings are commonplace in the care of frail older people [9]. Therefore, effective communication within and between services involved in the care of these patients is imperative [12]; providers require timely access to comprehensive, accurate, and up-to-date information about a person’s presentation, in a format that readily supports meaningful interpretation [13].

Written documentation and verbal handovers vary in quality and may fail to convey the complexities of an older adult’s condition, including functional capabilities and support needs. A growing body of literature suggests that the use of digital multimedia, such as photographs and videos, may help to enhance communication in patient care. A Cochrane review indicated that transmission of digital images between care providers may reduce time to commence treatment once a patient has presented to a service [14]. Moreover, a scoping review reported on the value of patient-generated photos and videos in helping professionals to obtain a more holistic understanding of patients, supporting diagnostic processes and monitoring of treatment outcomes, for example, documenting the healing of postoperative wounds [15]. Our recent systematic review synthesized empirical research evidence and professional and regulatory guidance on video recording patients for direct care purposes [16]. Review findings suggest that video recording patients to support care delivery is largely acceptable to patients, where privacy and data security risks are suitably mitigated [16].

Video recordings may support improvements in the safety and quality of care for older adults with frailty, in providing detailed visual information about a patient’s functional presentation, support needs, and care preferences. Nevertheless, ethical considerations warrant detailed examination prior to the routine use of video recordings within patient care [16]. Previously, a lack of secure and efficient ways to acquire, store, and share photographs and videos may in part explain why multimedia data have not been fully exploited in health care. Further research is required to explore the acceptability, feasibility, and potential value of video recordings that are securely embedded within the electronic patient record (EPR) for use in the care of older people living with frailty.

### Objectives

The primary objective of this study was (1) to determine the acceptability of using video recordings to capture the functional abilities, support needs, and preferences of older inpatients with frailty, to support clinical assessment and care delivery. Secondary objectives were (2) to explore the feasibility of implementing video-based patient records (the Isla Health Digital Pathway Platform) within the acute medical ward setting for older adults, and (3) to appraise the perceived effectiveness of patient videos for supporting care in this context. Study findings will be used to inform the decision-making about progression to a definitive trial, examining the effectiveness of patient video recordings for improving the quality and safety of care transitions.

## Methods

### Design

This study comprised a single-center, nonrandomized, mixed methods pilot study with an embedded process evaluation (ClinicalTrials.gov ID: NCT06504641) to inform decision-making about progression to a definitive trial to evaluate clinical impact. Where applicable, in line with guidance for nonrandomized pilot and feasibility research [17], the study reporting adheres to the CONSORT (Consolidated Standards of Reporting Trials) extension to pilot and feasibility trials [18], with intervention description informed by the Template for Intervention Description and Replication (TIDieR) guidelines [19].

### Setting and Participants

Participants were recruited from 3 acute medical wards for older adults within a large acute National Health Service (NHS) Trust in London, United Kingdom. We aimed to recruit 30 patient-carer dyads and 35 clinical staff members from the wards, according to the eligibility criteria defined in Table 1. Carers consisted of family members or friends who provided the patient with unpaid, informal care. Our sampling approach was designed to permit appraisal of acceptability and feasibility in principle, through a pragmatic yet purposively diverse sample of patients, carers, and ward staff.

**Table 1.** Overview of study eligibility criteria for the nonrandomized mixed methods pilot study involving older adult inpatients with frailty.^a^

Participants	Inclusion criteria	Exclusion criteria
Patients	Inpatient within acute medical wards for older adults during the 3-month intervention pilot phase Aged >65 years old Assessed as frail or prefrail by the direct care team Capacity to consent to participate OR lacked capacity to consent and a “personal consultee” was available to advise on patient’s likely wishes about participating	Lacked capacity to consent AND a “personal consultee” was not available to advise on the patients’ likely wishes about participating
Carers	Aged >18 years old Provided unpaid assistance to the patient for daily activities	Carers were excluded if the patient declined study participation
Ward staff	Clinical staff working on 1 of 3 participating acute medical wards for older adults Working regular shifts during the study initiation and 3-month pilot phases	Ad hoc bank or agency staff members Permanent staff members who are on long-term leave (eg, sickness and parental leave) during the 3-month pilot phase.

a Carers included family members and friends who provided the patient with unpaid, informal care.

### Intervention

The Isla for Frailty intervention provided a video-based patient record function via the web-based Isla Health Digital Pathway Platform, which interfaces with EPR systems such as Cerner. Isla Health is a technology company delivering a digital pathway platform for health care staff to support patients throughout their care journey. One of its features is a visual patient record, which enables health care staff to securely capture and review videos as part of a patient’s care. Video data are securely stored within encrypted cloud storage; health care staff can view these data within the EPR system, or directly via the Isla platform, using a secure weblink requiring the user’s NHS email address and password. To capture visual data using the Isla platform, the user must be logged in on a mobile device (eg, smartphone and tablet) with an in-built camera and Wi-Fi connection. The Isla Health Digital Pathway Platform is approved by NHS Digital and satisfies NHS data security and protection requirements. Wards were supplied with a designated tablet device to use for video capture.

Video recordings were captured to document aspects of a participating patient’s condition or functional ability considered by the direct care team as potentially useful in supporting clinical decision-making, care continuity, or multidisciplinary team (MDT) communication within the patient care journey. For example, videos might be used to capture a patient’s mobility and transfers, behavior, or patient preferences. Personal care or toileting was not recorded. Patient videos were viewable by the direct care team during the patient’s ward stay. Ward staff were encouraged to view and use videos in a way they felt was useful for supporting patient care, such as to support communication during shift handovers, to inform discharge planning, or in discussions with family members. The study protocol did not dictate when videos should be reviewed; therefore, the study examined the real-world use of patient videos within the acute older adult ward setting.

Participating staff had completed prior mandatory training in Data Security Awareness and were also trained in using the intervention prior to the 3-month pilot. Training emphasized the need to confirm participating patients’ consent prior to video capture and to stop recording if a patient showed any signs of distress (verbal or nonverbal) or if another patient entered the shot. Staff were advised to protect patient dignity, ensuring patients were appropriately dressed and drawing curtains around the bed space where required. Contact details for the study clinical lead were shared with all participating staff who wished to discuss any questions or concerns.

### Recruitment

#### Enrollment of Ward Staff

We engaged with staff working on the acute medical wards for older adults over a 2-month period (February 12-April 13, 2024) directly preceding the 3-month pilot phase for the video-based patient record intervention, inviting them to take part in the study. All clinical staff working on the 3 pilot wards received written and verbal information about the study aims, the intervention purpose, permitted uses of the video recordings, and information governance procedures. Staff were reassured that they could take part but opt not to be involved in video recording patients or appearing in patient videos themselves. They were also informed that they could choose not to participate in the evaluation of the video recording intervention, or to decline participation altogether without giving a reason. Staff who wished to take part were given opportunities to ask questions prior to providing their written informed consent.

#### Enrollment of Patients With Capacity to Consent

Patient screening and enrollment procedures are displayed in Multimedia Appendix 1. Over the 3-month intervention pilot phase (April 15-July 14, 2024), potential participants (inpatients on the 3 participating wards and their carers) were screened for eligibility by the direct care team, who explained the study purpose and provided patients and carers with participant information sheets. The direct care team flagged all potential participants to the researchers. Once the patient was clinically stable, a member of the research team visited the patient on the ward to confirm eligibility and complete enrollment procedures. Patients were offered opportunities to ask questions about the study and asked whether they might be interested in participating. Where required, easy-read participant information sheets were used to support patient understanding about what taking part would involve. Potential participants were offered additional time to decide whether they wished to take part. Where the direct care team reported concerns that a patient may lack capacity to consent to participation, the study team assessed and documented decision-specific capacity, considering whether the patient could understand what participation would involve, retain the study information, weigh this information to make a decision, and communicate their decision.

After consideration, patients who wished to participate and who had the capacity to consent were asked to sign a version of the consent form for patients with capacity. Patients were reassured that they could withdraw at any time. Within the consent process, patients were asked to consider whether they would like to remain in the study, should they lose capacity during the study period (eg, due to delirium onset). Patients were also asked whether they would still like their data to be used in the study. Participant information sheets contained a clear statement regarding retention and use of identifiable data following loss of capacity. Continued participation for such patients was subject to the same protocols for enrolling patients lacking capacity to consent, as detailed in the next section. During the study, the capacity of participants was monitored by the direct care team; the clinical lead communicated with the research team regularly about capacity concerns.

#### Enrollment of Patients Lacking Capacity to Consent

For patients assessed to lack the capacity to consent to participate, the research team still provided the potential participant with information about the study according to their level of understanding, using visual aids as needed (eg, the easy-read participant information sheets). In line with the requirements of Section 32(3) of the Mental Capacity Act 2005 and the Department of Health’s Guidance on nominating a consultee for research involving adults who lack capacity to consent (2008), reasonable steps were taken to identify a personal consultee for these patients [20,21]. Personal consultees were identified by the patient’s direct care team and included family members and carers (unpaid), who knew the patient well and who assisted them with their daily activities (eg, decisions about their welfare). Members of the ward staff team could not be nominated as personal consultees due to their own involvement in the study. Patients were excluded where no appropriate person could act as a personal consultee.

Potential personal consultees were approached first by the direct care team, who outlined the study purpose and the role of a consultee, emphasizing their right to decline acting as a consultee for the patient. For potential personal consultees who agreed to be contacted directly by the research team, a member of the research team then provided detailed written and verbal information about the study, including the consultee participant information sheet. The consultee was asked to consider the patient’s likely wishes about taking part. The research team made it clear to the consultee that they were not being asked to provide their personal views on study participation, nor to consent to the study on the patient’s behalf. Recruitment decisions were made by the research team in line with the consultee’s advice and any previous relevant statements or wishes communicated by the patient, whether verbal or nonverbal. Consultee advice was documented on a printed consultee declaration form or via an electronic version of the form. Where a consultee advised that the patient would not have wanted to take part, the researcher abided by this. Patients were also excluded if the consultee declined to offer advice about the patient’s likely wishes.

#### Enrollment of Carers

Carers of participating patients were also invited to take part in the evaluation during the 3-month pilot phase. Where carers were present while a member of the research team was on the ward, they were approached by the researcher accordingly. Otherwise, a ward team member contacted carers to ascertain whether they were happy to be contacted (via email or telephone) by a member of the research team. Carers who wished to participate after reviewing the carer participant information sheet were asked to sign a consent form for carers, via a printed version of the form, or an electronic form sent to the carer via email or text message. For all patients and carers, reasons for nonparticipation were documented within a screening and enrollment log.

### Data Collection

Study data sources are summarized in Table 2 and described below.

**Table 2.** Overview of outcomes, measures, and data sources for each objective of the nonrandomized mixed methods pilot study involving older adult inpatients with frailty.

Study objective, outcomes, and measures	Data source
Acceptability	
Patient recruitment and retention	
Percentage of eligible participants who were enrolled into the study	Screening and enrollment log
Percentage of eligible participants declining enrollment and reasons for nonparticipation	Screening and enrollment log
Video recording requests by care team	
Number of videos requested, attempted, and submitted	Video tracker
Suitability of patient videos	Video evaluation questionnaire
Desire to see more patient videos in the future	Video evaluation questionnaire
Perceived acceptability	
Patient and carer-reported acceptability	Semistructured interview at or within 2 weeks of discharge
Ward staff team-reported acceptability	Semistructured interview after the 3-month pilot
Feasibility	
Diversity of patient sample	
Patient clinical and demographic characteristics	Screening and enrollment log
Privacy and security concerns	
Number of videos raising cause for concern reported to the clinical lead	Video tracker
Use of the Isla Health Digital Pathway Platform	
Percentage of participants with one or more video linked to EPR^a^	Isla Health Digital Pathway Platform metadata
Reasons for unsuccessful attempts to take a video	Video tracker
Video view metrics	Isla Health Digital Pathway Platform metadata
Intervention barriers and facilitators	
Patient and carer-reported intervention barriers and facilitators	Semistructured interview after the 3-month pilot
Ward staff team-reported intervention barriers and facilitators	Semistructured interview after the 3-month pilot
Perceived effectiveness	
Perceived impacts on: assessment and clinical decision-making; multidisciplinary team communication; care continuity during a hospital stay; person-centered care during a hospital stay	
Potential usefulness of intervention to ward staff team	Video evaluation questionnaire
Ward staff team-reported perspectives on intervention impacts	Semistructured interview after the 3-month pilot
Patient and carer-reported perspectives on intervention impacts	Semistructured interview at or within 2 weeks of discharge

a EPR: electronic patient record.

#### Screening and Enrollment Log

The screening and enrollment log was used to document patient recruitment status, demographic characteristics, and, where applicable, reasons for nonparticipation. A paper version of the log was initially populated. It was then digitized at regular intervals within the 3-month intervention pilot phase and stored in Imperial College Healthcare NHS Trust’s iCARE secure data environment.

#### Video Tracker

A video tracker spreadsheet (Microsoft Excel) was developed to document patient video requests, attempts, submissions, and any issues associated with taking the recordings. The video tracker also captured information about the aspect of a participating patient’s care or functional abilities to be video recorded. For example, video foci may include a person’s mobility baseline or their support needs when eating and drinking.

#### Video Evaluation Questionnaires

Staff experience of viewing patient videos was appraised using a brief, anonymous, paper-based video evaluation questionnaire, consisting of closed-ended, multiple-choice questions with space for free-text comments (Multimedia Appendix 2). Participating staff were asked to complete the questionnaire for each video they viewed. At the end of the 3-month pilot, questionnaire data were digitized (Microsoft Excel) and stored securely within the iCARE secure data environment.

#### Interviews With Patients and Carers

Patients with the capacity to consent to participation and carers were invited to take part in brief, semistructured interviews at or within 2 weeks of hospital discharge. Consent to take part in an optional interview was provided during the enrollment phase. All patients with the capacity to participate in an interview who had not declined the optional interview component at enrollment were invited to take part as they approached hospital discharge. Likewise, all participating carers were contacted to arrange the optional interview, unless they had previously declined at enrollment. Where the direct care team or research team felt it appropriate, patient capacity was assessed again prior to undertaking the interview, due to the potential for fluctuating capacity in this group. Patients and carers were asked to verbally confirm their consent prior to starting the interview. Interviews were conducted on the ward prior to discharge, or via telephone where patients had already been discharged.

Interviews were conducted by a member of the research team (PA or RO) using a topic guide for patients and carers (Multimedia Appendix 3), seeking to understand patients’ experiences of being video recorded. Researchers adapted their language to the patient’s level of understanding to enable patients with cognitive impairment to participate. Patient and carer interviews were completed by August 14, 2024, reflecting the date of discharge of the final patient who was eligible to take part in an interview. Interviews were audio-recorded and professionally transcribed. Names and identifiers were replaced with pseudonyms during the transcription process. Transcripts were transferred to and stored within the iCARE secure data environment.

#### Interviews With Ward Staff

Semistructured interviews with consenting staff members were undertaken within 2 months of the end of the 3-month intervention pilot phase. All participating staff members were approached to take part in an interview, unless they had declined this optional study component at the point of enrollment. Interviews used a version of the topic guide for staff and explored their experiences with the video-based patient records (the Isla Health Digital Pathway Platform) and perceived impacts on patient assessment and clinical decision-making; team communication; and care delivery (Multimedia Appendix 4). Audio-recorded interviews were conducted in a private space on or near the ward, or via telephone according to interviewee preference, with the final interview taking place on October 3, 2024. Written consent for the interviews was documented at recruitment. Names and identifiers were replaced with pseudonyms during the transcription process. Transcripts were transferred to and stored within the iCARE secure data environment.

#### Isla Health Digital Pathway Platform Metadata

At the end of the 3-month video-based patient record pilot (the Isla Health Digital Pathway Platform), video view metrics were exported from the Isla audit log. The anonymized video view metrics spreadsheet was transferred to and stored within the iCARE secure data environment.

### Outcomes

In line with study objectives, the primary outcome we sought to assess was patient, carer, and ward staff team perspectives on the acceptability of the video-based patient records (the Isla Health Digital Pathway Platform). Use of the Isla platform and intervention barriers and facilitators were among secondary outcomes measured to assess the feasibility of implementing video-based patient records within the acute medical inpatient setting for older adults. Finally, 4 outcomes, including perceived impacts on care continuity in the hospital, were appraised to examine the perceived effectiveness of video-based patient records for supporting older adult inpatient care. Outcome measures and data sources for each objective are summarized in Table 2.

### Data Analysis

Using Microsoft Excel, descriptive statistics were computed to summarize numerical data as to participant recruitment and retention rates; proportions of patients with videos linked to the EPR; video view metrics; and numbers of videos raising cause for concern. Frequencies and percentages were calculated for categorical variables, with means and SDs presented for continuous variables. Framework Analysis, following the 5-step approach described by Ritchie and Spencer, was applied to qualitative study data [22]. Accordingly, following a phase of familiarization with interview transcripts and free-text video evaluation questionnaire data, key concepts and ideas were noted by 1 author (PA) and discussed iteratively with 2 further authors (RO and AT), to interrogate immediate observations and assumptions about these data. At this stage, the researcher’s analytical observations were used together with the Theoretical Framework of Acceptability of Healthcare Interventions by Sekhon to shape an initial thematic framework [23], combining a priori concepts with inductively generated codes. The Theoretical Framework of Acceptability of Healthcare Interventions comprises 7 constructs for appraising intervention acceptability: affective attitude, burden, ethicality, intervention coherence, opportunity costs, perceived effectiveness, and self-efficacy. Component constructs are defined in Multimedia Appendix 5. The derived thematic framework was then applied to all transcripts by 2 authors (PA and RO), with regular collaborative coding meetings convened by coders to appraise consistency and appropriateness in use of the framework. Data were then charted into a framework matrix for review and interpretation (PA, RO, and AT).

### Information Power

Adequacy of the participant sample and subsequent data collection were appraised on an ongoing basis according to the 5 dimensions of “information power” [24]. Several qualities of the study suggested that a less extensive sample would be required to achieve sufficient information power. First, our a priori study objectives were narrowly focused on the acceptability, feasibility, and perceived effectiveness of a specific intervention. Second, the study eligibility criteria ensured dense sample specificity, where participants all held characteristics which were highly specific to study objectives (eg, patients who were older adults with frailty admitted to 1 of 3 pilot study wards, carers who were unpaid carers to enrolled patients, and ward staff who were clinical staff employed on a permanent basis on one of the pilot study wards). Third, our primary objective to examine intervention acceptability was underpinned by established theory, in the form of the Theoretical Framework of Acceptability of Healthcare Interventions by Sekhon [23]. A fourth dimension, concerning the quality of dialogue, could only be assessed during the data collection phase, rather than at the study outset. We therefore reflect on this later in the paper. Finally, since Framework Analysis approaches entail both within- and between-case analysis using the derived framework matrix, we deemed that a larger volume of data would be necessary to evaluate study objectives, relative to a solely within-case analysis.

### Patient and Public Involvement and Engagement

Patient and public involvement and engagement (PPIE) was central to this research. A member of the public with lived experience of caring for an older family member with frailty joined the team as a PPIE researcher, developing a storyboard characterizing patient experiences of frailty and discontinuity at care transitions. This was used to engage ward staff members as to the potential value of the research, helping to support successful staff enrollment. Contributions to data analysis, interpretation, and coauthoring this paper ensured that the perspectives of patients and carers informed each stage of the study.

### Ethical Considerations

Ethics approval was granted by an English NHS research ethics committee (IRAS ID: 313814). Informed consent was obtained from all participating carers and ward staff members via completion of paper or electronic consent forms. For patients with the capacity to consent to participation, informed consent was documented by signing a paper consent form. Personal consultees, who knew the patient well and assisted them with their daily activities, were asked to consider the patient’s likely wishes about taking part for those patients assessed to lack the capacity to consent to participation. Consultee advice was documented on a printed or electronic consultee declaration form. Enrollment and consent procedures are described in detail in the “Recruitment” section. Participant names and identifiers were replaced with pseudonyms and all study data were securely stored within Imperial College Healthcare NHS Trust’s iCARE secure data environment. Further details are provided within the “Data Collection” section. Research participants did not receive payments or other compensation to take part.

## Results

### Overview

We recruited 107 study participants, consisting of 58 ward staff team members, 29 patients, and 20 carers. Twelve carers additionally acted as a personal consultee for recruited patients. Participating staff included registered nurses and nursing assistants (36/58, 62.1%); physiotherapists, occupational therapists, and therapy assistants (9/58, 15.5%); doctors (11/58, 19.0%); and administrative staff (2/58, 3.4%). Table 3 shows the baseline characteristics for patients who were invited to participate, by enrollment status. Video evaluation questionnaires were completed by just 4 staff members who had watched one or more patient videos (2 nurses and 2 therapy professionals), owing to issues with interpreting Isla Health Digital Pathway Platform metadata, detailed below alongside feasibility findings. Postintervention interviews were conducted with 70 participants, comprising 10 patients, 16 carers, and 44 staff members and lasted between 2 and 25 minutes (mean 11, SD 4.7 minutes).

**Table 3.** Baseline characteristics of older adult inpatients with frailty who were invited to participate by enrollment status.^a^

Characteristics	Enrolled patients (n=29)	Nonenrolled patients (n=22)
Sex, n (%)		
Male	18 (62.1)	12 (54.5)
Age (years), mean (range)	82.9 (72-96)	82.2 (66-92)
Ethnicity, n (%)^b^		
White	14 (48.3)	13 (59.1)
Black, Black British, Caribbean or African	3 (10.3)	2 (9.1)
Asian or Asian British	2 (6.9)	3 (13.6)
Mixed or Multiple Ethnic Groups	0 (0)	1 (4.5)
Other	8 (27.6)	2 (9.1)
Not stated	2 (6.9)	1 (4.5)
Dementia diagnosis, n (%)		
Diagnosis received	10 (34.5)	4 (18.2)
Main language spoken at home, n (%)		
English	27 (93.1)	18 (81.8)
Clinical Frailty Scale, n (%)		
1: Very fit	0 (0)	0 (0)
2: Well	0 (0)	0 (0)
3: Managing well	0 (0)	0 (0)
4: Vulnerable	0 (0)	2 (9.1)
5: Mildly frail	5 (17.2)	3 (13.6)
6: Moderately frail	8 (27.6)	4 (18.2)
7: Severely frail	16 (55.2)	13 (59.1)
8: Very severely frail	0 (0)	0 (0)
9: Terminally ill	0 (0)	0 (0)

a Other languages spoken at home included Urdu, Gujarati, Greek, and Kurdish.

b Ethnicity categories were defined according to UK Census classifications. White includes English, Welsh, Scottish, Northern Irish or British, Irish, Gypsy or Irish Traveller, Roma, and any other White background. Black, Black British, Caribbean or African includes Caribbean, African, and any other Black, Black British, or Caribbean background. Asian or Asian British includes Indian, Pakistani, Bangladeshi, Chinese, and any other Asian background. Mixed or Multiple Ethnic Groups includes White and Black Caribbean, White and Black African, White and Asian, and any other Mixed or multiple ethnic background. Other includes Arab, and any other ethnic group.

### Acceptability

#### Patient Recruitment and Retention

**Figure 1.**
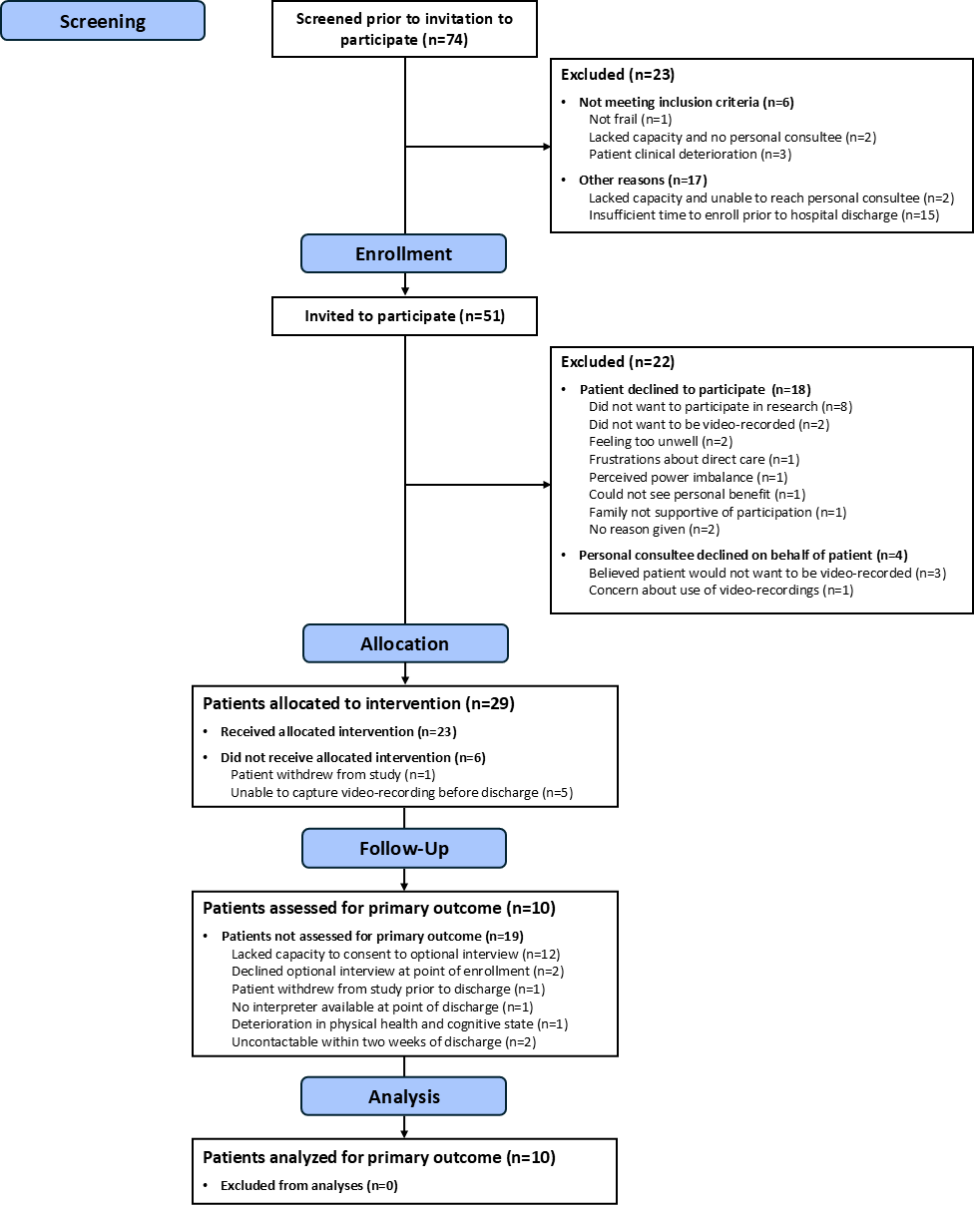
Patient flow throughout the nonrandomized mixed methods pilot study (modified from CONSORT [25]). CONSORT: Consolidated Standards of Reporting Trials.

Fifty-one patients were invited to participate, of whom 29 patients (56.9%) were recruited: 17 (58.6%) patients gave informed consent, while a personal consultee was involved in the decision for 12 (41.4%) further patients (Figure 1). One patient withdrew from the study prior to receiving the intervention. Among those who declined participation (22/51, 43.1%), key reasons given by patients included not wanting to take part in research (n=8), not wanting to be video-recorded (n=2), or feeling too unwell (n=2). Consultees who declined on behalf of a patient did so due to believing that the patient would not like to be video recorded (n=3), or concerns about how video recordings would be used (n=1).

Only patients with the capacity to provide their own consent to participate were invited to take part in an interview to assess outcomes. Participating patients were not excluded from interviewing based on whether they had personally received the allocated intervention during the pilot, defined as having at least one video linked to the EPR prior to hospital discharge. As such, follow-up status is displayed for all patients initially enrolled to the study.

#### Video Recording Requests by Care Team

During the 3-month intervention pilot, 44 videos were requested by ward staff, with 37 video recordings attempted and 36 submitted within the EPR. Video recordings were focused on patient transfers (n=16), other aspects of mobility (n=13), eating and drinking support (n=3), and patient behavior (n=2). Further videos documented patient-staff interactions (n=1) and seizures (n=1). Video evaluation questionnaire data indicated the suitability of patient videos and the desire among those staff to see more videos in the future. Indeed, all 4 respondents endorsed that videos were of suitable length and quality for clinical interpretation, expressing a desire to see more patient videos in the future.

#### Perceived Acceptability

##### Overview

**Figure 2.**
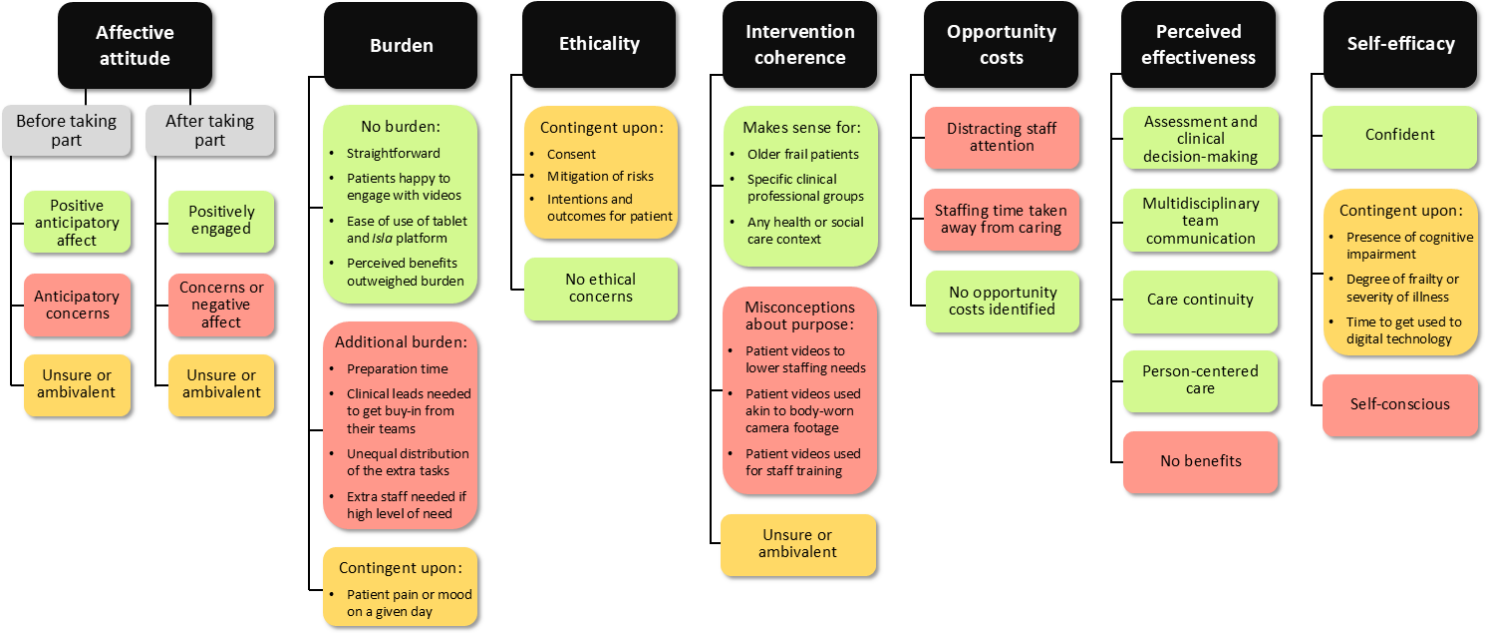
Overview of nonrandomized mixed methods pilot study findings about the acceptability of using video recordings as part of older adult inpatient care.

Interviewed patients, carers, and ward staff alike (66/70, 94.3%) typically rated video-based patient records as “completely acceptable” or “acceptable.” Three participants stated that they had “no opinion” (4.3%), while a single patient felt that the intervention was “unacceptable” (1.4%); no explanation was provided for this rating. Detailed qualitative findings as to the acceptability of video-based patient records, informed by the Theoretical Framework of Acceptability of Healthcare Interventions by Sekhon, are presented as follows and summarized in Figure 2 [23].

##### Affective Attitude

Reflecting on how they felt about the intervention prior to participating, those with positive anticipatory affect gave several reasons for this perspective. Video-based patient records were expected to help in establishing an individual’s baseline function upon admission, allowing patients and staff alike to appraise progress during the inpatient stay. Staff believed the intervention could foster improved MDT communication and clinical decision-making, valuing the opportunity to pilot the intervention prior to decisions about rollout into routine care:

*We get new things turn up all the time, “Oh we are doing this now, we got this new policy”, whereas because it’s being studied in a research way there is going to be lots of oversight, consent, and feeding back.*

[Doctor]

Anticipatory concerns were about information security and maintaining patient dignity, particularly where patients lacked the capacity to personally consent to taking part. Staff were also apprehensive about potential workload impacts and whether patients and families would be supportive of the intervention. In practice, capturing and viewing patient videos was less burdensome than expected and staff were surprised to find that many patients were enthusiastic about taking part:

*I was a bit skeptical, but found that the more we saw the patients, the more eager they were about the recording.*

[Therapies staff]

##### Burden

Patients and carers believed that burdens to patients in participating in the intervention were limited:

*I felt it was something that needed to be done, it didn’t take very long. I didn’t have a lot to do so it was very positive.*

[Patient]

The potential to exacerbate behavioral symptoms in patients with agitation or cognitive impairment was carefully considered by staff and carers. This was mitigated by avoiding filming or terminating patient videos immediately upon any indication of patient distress (verbal or nonverbal). Burdens for staff included time required for preparation or additional staffing needs where patients required assistance of 2 for mobilization, with a third staff member needed to capture the video.

##### Ethicality

Participants regarded the use of video recordings within the assessment and care of older inpatients with frailty as ethical, on the grounds that certain conditions were met. A central view was that ethicality was contingent upon patient consent to appear in videos. Where a person was assessed to lack the capacity to consent, differing views were presented as to whether a personal consultee or next-of-kin should be allowed to make this decision on a patient’s behalf. Given that a person’s next-of-kin would typically be best positioned to understand the patient’s likely wishes, next-of-kin consent was largely considered suitable. However, others voiced that patient consent was the only basis on which the intervention could be judged as ethical:

*I would feel something like that needs to come from them [patients]… and I don’t think the next-of-kin should be making that decision for them.*

[Carer]

Mitigation of risks associated with video capture was another prerequisite for ethicality. A central principle was that a patient’s dignity must always be upheld during video recording, particularly given the permanence of digital patient records. Patient confidentiality and information security were further risks agreed by participants to warrant safeguards. Participants described feeling reassured about the extensive safeguards in place throughout the intervention pilot. There was consensus that patient videos should be viewed only by staff members who need to see them and must be stored in a secure digital environment that is resilient to cyberattacks.

The intentions and outcomes of the use of video-based patient records comprised another determinant of their ethicality. Where an individual’s personal, religious, and cultural beliefs were considered and there was a clear clinical rationale for capturing a patient video to optimize an aspect of care, conditions for ethicality were met. Indeed, staff indicated that patient videos may help to accelerate processes of identifying discharge placements for patients, thus reducing other risks associated with lengthy hospital stays, including deconditioning or exposure to hospital-acquired infections. There was a perception that withholding such an intervention from patients, due to concerns about frailty or cognitive impairment, meant that older inpatients risked missing out on potentially enhanced care. Patients justified ethicality based on enjoying tracking their own progress and experiencing no negative outcomes from taking part:

*It hasn’t hurt me, so why should I complain?*

[Patient]

Beneficial patient outcomes associated with video-based patient records were thought most likely to be realized where there is potential for an individual to return to their mobility baseline, while benefits were deemed less clear for extremely frail or cognitively impaired patients.

##### Intervention Coherence

It was evident from some participants’ accounts that they understood how video-based patient records could support patient care and why the intervention had been piloted within the 3 wards. Those who could confidently explain the intervention aims felt it made particular sense to use patient videos in the care of older patients, where rapid changes in presentation may occur:

*I can see now how it might be important for people to see the transformation in me, the way I was a few days ago. Now, I’ve lost ten years.*

[Patient]

Comparisons to usual care were made at several points. Staff described the limitations inherent in text-based patient information and reflected upon the potential added value of visual data for obtaining insights into a patient’s needs:

*It’s quite good technology to have, like I say, written documentation doesn’t always convey what’s going on, the patient’s abilities. So, to actually see something, especially in regards to moving and handling, things like that.*

[Nursing staff]

Staff believed that patient videos had applications for all MDT members. However, the intervention was thought most relevant to the work of physiotherapists and occupational therapists, as well as doctors. Moreover, others regarded that video recordings may be coherently used in any health or social care context.

In contrast, some patients and carers were unsure about how the intervention could be useful as part of clinical care. Furthermore, several misconceptions were expressed about the purpose of patient videos. Some staff members believed that patient videos would be used to reduce staffing requirements. Likewise, others misunderstood how and why patients may be filmed, believing that video capture would be used in the same way as body-worn cameras, which are typically used for continuous or targeted filming to document violence and aggression in health care or policing contexts.

##### Opportunity Costs

Opportunity costs, where other potential benefits were forgone in implementing the video-based patient record intervention, were seldom identified. A concern was that in capturing patient videos, staff may be distracted from risks on the ward, such as wandering patients:

*If a patient fell… you obviously want to focus your attention on what’s going on. You don’t want to be thinking about, “Is that angle right? Am I capturing everything?”*

[Therapies staff]

Second, an additional member of staff was often asked to assist in holding the tablet during video capture, thus stepping away from the task they were completing at the time.

##### Self-Efficacy

Finally, many participants expressed confidence in either capturing (staff) or appearing in videos (patients, carers, or staff). Self-efficacy was reportedly lower where patients had cognitive impairment; patients were also less comfortable appearing in videos on days when they were especially unwell. Some ward staff indicated initial hesitancy in using a new form of digital technology, needing time to become accustomed to using the tablet. A minority of patients and staff felt self-conscious over their physical appearance or about the sound of their voice when appearing in videos.

### Feasibility

#### Diversity of Patient Sample

Enrolled patients were largely male (18/29, 62.1%), had a mean age of 82.9 (range 72‐96, SD 6.7) years, mostly spoke English at home (27/29, 93%), and were diverse in ethnicity (Table 3). Clinical Frailty Scale scores for participating patients ranged from 5 (Mildly frail) to 7 (Severely frail), with most patients evaluated to be “Severely frail” (n=16) [26]. Around one-third (10/29, 34.5%) had a dementia diagnosis. Baseline characteristics were similar between enrolled and nonenrolled patients, apart from dementia diagnosis and main language spoken at home, where higher proportions of participating patients had dementia and spoke English.

#### Privacy and Security Concerns

During the 3-month pilot, the study clinical lead was asked to review a single patient video owing to privacy concerns. In the video, another patient could be observed stepping into the video background. The video was removed from the Isla Health Digital Pathway Platform, owing to concerns that the other patient might be identifiable from the footage.

#### Use of the Isla Health Digital Pathway Platform

Videos were captured on a total of 19 days within the 3-month pilot. The video recording process typically involves 2 or 3 staff members, with one to carry out the recording and others to provide direct support to the patient. Information as to those involved in this process was limited to the person capturing the video recording only, based on login data for the platform. We ascertained that 11 different staff members used their logins to capture and upload videos but were unable to determine the total number of staff members involved in the recording process in a supporting role.

Use of the Isla Health Digital Platform Pathway was similar across the 3 pilot wards. Ward 1 captured 13 videos (among n=10 recruited patients), with 17 captured on Ward 2 (among n=13 recruited patients), and 6 on Ward 3 (among n=6 recruited patients). Overall, of 29 participating patients, 79.3% (n=23) had at least one video linked to their EPR at the point of discharge. The research team was notified of 12 unsuccessful attempts to take a video. Primary reasons documented included the patient declining (n=4), patient cognitive or medical deterioration (n=3), and insufficient staffing (n=2).

Video views by ward staff were quantified through Isla Health Digital Pathway Platform metadata, where staff clicked to view and expand a video tile, resulting in a documented “View submission” action. Four such views were counted. However, toward the end of the pilot phase, we ascertained through observing staff interact with the platform that watching a video without expanding on the video tile was instead recorded within the Isla platform metadata as “View folder.” Accordingly, excluding cases where a “View folder” action occurred directly before or after the same staff member uploaded a video for a given patient (when the folder view likely served a different purpose), we estimate that there were 17 video views during the study. Seemingly low video view rates among staff were corroborated by anecdotal insights obtained through staff interviews. Although staff members could typically identify potential intervention benefits at the team level, when asked directly, interviewed staff commonly attested that they had not personally viewed or sought to access a patient video at any point during the 3-month pilot.

#### Intervention Barriers and Facilitators

**Figure 3.**
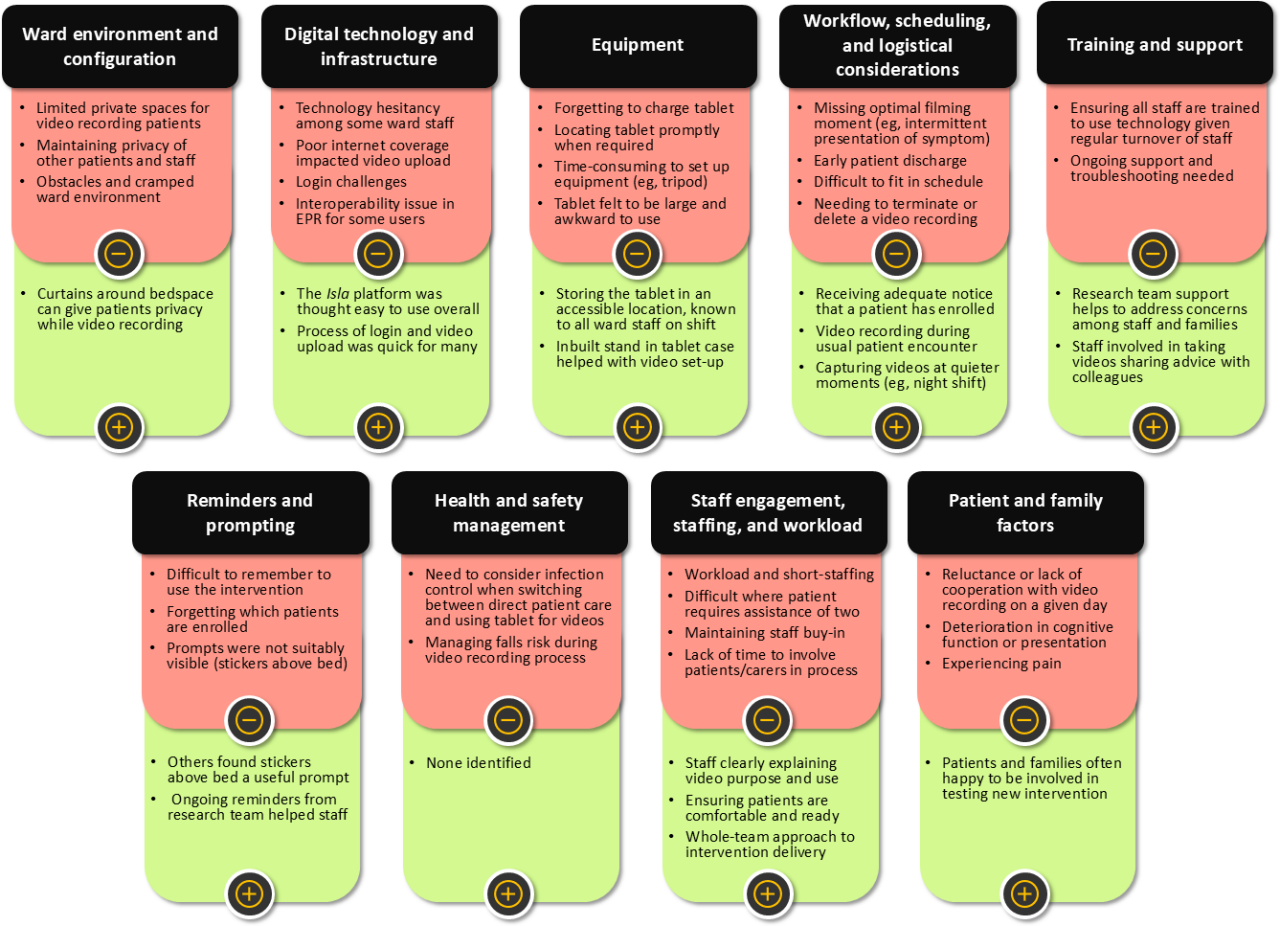
Barriers and facilitators to implementing video-based patient records in acute medical wards for older adult inpatients with frailty. EPR: electronic patient record.

Nine themes were inductively developed from interview data as to the barriers and facilitators to implementing the video-based patient records intervention in the care of older adult hospital inpatients with frailty (Figure 3).

##### Ward Environment and Configuration

The crowded, busy ward environment and layout were such that staff struggled to capture patient videos while maintaining the privacy of participating patients. Likewise, difficulties were faced in ensuring that other patients and staff members were not captured in the background of patient videos. Curtains around the bed space were valued by staff for facilitating a degree of privacy during the video recording process.

##### Digital Technology and Infrastructure

Digital technology and infrastructure barriers stemmed from poor quality Wi-Fi coverage across the 3 pilot wards. Technology hesitancy and delays or challenges in logging into the video-based patient records system were also experienced by some staff members:

*We are so tied down by logins, governance, two-factor authentication that if you want people to be able to have a low threshold to record, check, share something, you can’t do it.*

[Doctor]

Overall, the Isla Health Digital Pathway Platform was, however, considered straightforward to use.

##### Equipment

Staff reported several equipment-related challenges. Locating the designated tablet promptly when required or finding that the battery had not been charged by previous users were key frustrations. An inbuilt stand within the tablet case served as a facilitator to the video recording process, allowing staff to position the tablet on a surface within the ward (eg, bedside table) when another staff member could not assist with video capture.

##### Workflow, Scheduling, and Logistical Considerations

Further obstacles to using the intervention are related to ward workflow and logistical considerations. Videos often needed to be taken at an optimal moment when seeking to capture intermittent presentations (eg, seizures) or for fatigued patients. Patients were sometimes discharged earlier than expected, meaning that video recordings could not be captured in time or used to realize potential benefits. Staff described integrating video capture into their usual planned therapy sessions with participating patients, or capturing videos during quieter periods, to maximize efficiency and feasibility.

##### Training and Support

Regular staff turnover presented difficulties in ensuring all staff were trained in using the intervention. From the staff engagement and enrollment phase through to the end of the 3-month pilot, there were rotations in both junior doctors and specialty trainees on a national level. When asked about low apparent usage of videos, some staff members indicated that they could not recall how to access videos within the EPR:

*I think it’s quite straightforward when you record it, but I think there is still some sort of gap, like how you follow up with Isla outside of the iPad [tablet].*

[Therapies staff]

Nevertheless, staff and patients alike appreciated access to ongoing training and support from the research team throughout the 3-month trial period.

##### Reminders and Prompting

Remembering to capture and use videos within patient care was a persistent challenge. This served as a barrier to uptake and achieving a meaningful intervention “dosage” for participants, where it was felt that more videos throughout an individual’s inpatient stay would be necessary to visually demonstrate improvement or deterioration.

*I was naively hoping it would be more visible. Having people nudging and reminding definitely helps, but that would be my concern for wider adoption.*

[Doctor]

For other staff, there appeared to be low impetus to access and use patient videos once captured:

*Not many people would go and check, or would say, “Okay, on Cerner [EPR], I want to go and see that video.”*

[Therapies staff]

Reminders used throughout the pilot study period (eg, stickers above beds of enrolled patients and reminders from the research team) were valued but thought insufficient for comprehensive implementation.

##### Health and Safety Management

Managing safety when capturing videos, including staff’s ability to prevent patient falls during video-recorded mobility assessments, was an important consideration. Other challenges included infection control considerations when switching between delivering direct patient care and using the tablet to capture a patient video.

*I got pointed out for infection control… they recommended me leaving the iPad [tablet] there, going to wash my hands, come back and go onto the tablet. But I can’t leave the patient.*

[Therapies staff]

Staff detailed practical issues in capturing videos, where handwashing was required between each activity.

##### Staff Engagement, Staffing, and Workload

Moreover, intervention feasibility was also contingent upon staff engagement, staffing, and workload factors. Barriers were presented when wards were short-staffed, resulting in high workload for clinical teams and limited spare capacity to assist with capturing videos. Reassuring staff that video recordings were not intended to be used for appraising staff practice and recognizing the efforts of staff to deliver the intervention were important for maintaining staff engagement. Implementation was reportedly best where an MDT approach to capturing patient videos was adopted, so that individual professional groups (ie, nursing or therapy staff) did not feel alone in shouldering additional task burdens. Likewise, carers emphasized the importance of staff taking time to explain to patients why they wished to capture a video of a given activity or symptom and how the video may be used as part of their care:

*Is there rapport being built up? Is the person comfortable with those particular healthcare professionals?*

[Carer]

By allowing time to develop positive staff-patient relationships, patients felt more comfortable with taking part.

##### Patient and Family Factors

Finally, patient and family factors were important to the implementation of video-based patient records. On some days, interviewees described that patients were reluctant to accept any care or interventions on the ward, including patient videos. Older, frail patients may decline to engage owing to experiencing pain or fatigue on a given day. Seemingly uncooperative behavior was displayed at times among participating patients due to confusion or fluctuation in cognitive function, presenting a barrier to delivering the video-based patient record intervention:

*When they were trying to video something more positive with him, he decided not to be as positive! But that’s hit and miss with any of the patients I would think.*

[Carer]

Nevertheless, a key facilitator indicated by all participant groups was that patients and carers were often happy to be involved in trialing a new intervention which may improve their own care or that of others.

### Perceived Effectiveness

#### Overview

Perceived effectiveness of the video-based patient records intervention was appraised according to ward staff views on its potential usefulness in the care of older adult inpatients with frailty, as well as patient, carer, and staff perspectives on intervention impacts. Participants conceived of multiple ways in which video-based patient records could be potentially effective for use in the present context. Some participants were unaware of any intervention impacts. Such patients and carers described a lack of follow-up from ward staff about their videos, meaning that they were unsure whether the video recordings had been beneficial. No adverse events were reported. Study data indicated several ways in which video recordings may improve aspects of patient care.

#### Assessment and Clinical Decision-Making

Data from the 4 completed video evaluation questionnaires showed that ward staff considered the video they had watched was “very useful” for supporting patient assessment and clinical decision-making. Interviewees elaborated on the potential for patient videos to improve assessment quality by mitigating subjectivity in clinical interpretation and speeding up the assessment process. For behaviors or symptoms occurring at unpredictable intervals, videos also permitted staff to obtain specialist advice from other teams:

*It helped our neuro team see a patient who was having intermittent seizures… they [neurology team] would not have been able to see it otherwise.*

[Nursing staff]

Further potential benefits were in offering clear information about an individual’s care and support needs, providing evidence for referrals to other care providers. Staff reflected on challenges faced in securing onward care placements for patients who had exhibited fluctuating behavior and nursing care requirements in the hospital. Rehabilitation settings and nursing homes reportedly declined referrals or overestimated staffing costs necessary to provide patient care, based on patients having had an acute or episodic change in presentation (eg, delirium onset) during their admission:

*If we had someone come in for assessments, we had this evidence to say “Okay, look, on a good day this patient did it this way, so you need to assess them based on that*.”

[Nursing staff]

In contrast, an alternative position was that videos merely provided a snapshot of a person’s presentation:

*You’re putting someone into a slightly odd position saying, “Now, walk for us.” And I think you’ll get the Charlie Chaplin effect, people starting to do things that maybe they wouldn’t normally do… I think a camera is not always going to bring out the truth in people.*

[Carer]

Therefore, the potential for video-based patient records to improve assessment and decision-making precision was questioned.

#### Multidisciplinary Team Communication

All 4 staff members who completed video evaluation questionnaires reported that the video they had watched was “very useful” for communicating patient information to colleagues. The added communicative value of visual data over written documentation or verbal handovers was apparent from interview data. Carers were similarly supportive of patient videos, where their use might improve communication quality and concision:

*I don’t know whether that would completely eradicate the need for discussion… but it could perhaps enhance that, if there’s something that someone needs to show someone else.*

[Carer]

Participants also envisaged potential value for strengthening communication with other clinical settings. Carers felt that videos could optimize care timeliness and continuity by enabling remote communication with other care providers. This view was supported by staff, who also believed that patient videos may improve collaboration between clinical services, potentially reducing duplication of clinical assessments:

*We have a deficit of trust across boundaries… but if you see a video they know we are not making it up.*

[Doctor]

Moreover, videos were reportedly used to enhance communication with patients’ families:

*Sometimes the family says, “Oh, he could not do it at home.” When they see the patient sitting out and walking, it’s amazing them.*

[Nursing staff]

As such, this helped to provide a tangible demonstration of care and interventions received in the hospital and apparent impacts.

#### Care Continuity

Study findings suggest that video-based patient records could improve care quality by optimizing continuity, both within and beyond an individual’s hospital stay. Patient videos may support joined-up care by helping to establish a shared understanding of the goals of a patient’s admission, informed by videos showing their functional baseline. Similarly, participants reported that videos allowed staff to objectively monitor patients over time, facilitating greater awareness of signs of improvement or deterioration:

*For the patient it’s better because we know their progress… I’m video-recording to compare. We see the difference from bedbound to mobilizing.*

[Nursing staff]

Video-based patient records were also deemed valuable for aiding safe care transitions. Staff thought patient videos could assist community therapy teams to provide joined-up care following discharge. Since frail older adults often experience multiple hospital admissions, participants also envisaged using patient videos to gain insights into a person’s presentation during prior inpatient stays.

#### Person-Centered Care

The potential for video-based patient records to improve person-centered care was a prominent theme. By providing a visual record of a patient’s achievements throughout their hospital admission, patient videos promoted a focus on an individual’s goals and needs, contributing to more personalized care experiences:

*I was proud to see me in the video and pleased that I have made so much progress… it gave me great spirits.*

[Patient]

Moreover, the use of patient videos was thought to improve the quality of staff-patient engagement. Staff described how the process of delivering the intervention offered opportunities for patients to receive additional encouragement from ward staff, which in turn improved staff satisfaction:

*The patient was eager, because he was walking and the staff… they were following and cheering him on. It was so beautiful – he was able to mobilize himself. They were all so happy, the staff and the patient themself [sic] because he wanted to go home!*

[Nursing staff]

Finally, there was a sense that patients appreciated the choice over whether to take part in the intervention:

*It gives the patient a little say in what’s going on in their life as a patient.*

[Patient]

Where patients may feel they have limited involvement in care decision-making when in hospital, the opportunity to make choices about their care appeared to instill a sense of agency and empowerment.

## Discussion

### Principal Findings

Findings from this nonrandomized mixed methods pilot study suggest that the use of video recordings within patient assessment and care delivery is largely acceptable to older patients with frailty, their family members or carers, and clinical staff. This was demonstrated by promising participant recruitment and retention rates; enrollment targets for patients were almost met, while ward staff recruitment vastly exceeded intended numbers. The range of videos captured, from documentation of seizures through to establishing a person’s mobility baseline, shows that staff discerned multiple use cases for patient videos. On the grounds that ethical preconditions are met, including mitigation of concerns about information security and patient dignity, video-based patient records were largely deemed to be acceptable.

A secondary objective to explore the feasibility of implementing video-based patient records within acute wards for older adults revealed mixed findings. Diversity of the patient sample in terms of clinical and demographic characteristics was encouraging, indicating that risks of widening health inequalities through inequitable uptake are low, should the intervention be implemented into routine care. Implementation barriers were multiple, yet participants described a range of facilitators that supported successful delivery. However, video view metric estimates and anecdotal evidence from interviews with ward staff suggested limited engagement with videos once captured, thus casting doubt on the feasibility of attaining sufficient uptake among staff. We estimate that over half of all videos captured were never viewed. Nevertheless, patient, carer, and clinical opinion as to perceived intervention effectiveness yielded evidence of promise. Videos were considered useful in supporting clinical assessment, enhancing MDT communication, strengthening care continuity, and promoting person-centered care.

### Comparison With Wider Literature

Our findings are largely in accordance with the conclusions of a recent systematic review, which examined empirical research and regulatory guidance on the generation and use of video recordings as part of patient care [16]. Indeed, similarly to existing studies [16,27,28], we observed that video-based patient records were acceptable to patients, carers, and clinical staff alike, so long as ethical conditions were satisfied around upholding patient dignity and maintaining the security of these digital data [29]. Likewise, based on clinical opinion, we found that patient videos may aid the monitoring of care delivery and outcomes over time, aligning with the conclusions of comparable research [15].

Perhaps most striking was our finding that video-based patient records appear to be valuable for promoting person-centered care within the acute hospital setting. The intervention seemed to instill a sense of agency and empowerment in older adults with frailty who took part in video recordings. Staff experienced secondhand satisfaction upon observing a patient demonstrating a functional improvement, such as walking unaided, when they had previously felt too frail to do so. This finding resonates with prior research from physiotherapy and rehabilitation contexts, where video recordings documenting improvements in patient gait throughout their inpatient stay were found to improve patient motivation and satisfaction [30]. Likewise, although we piloted the use of patient videos captured by ward staff, rather than exploring patient-generated visual media as reported within a further review [15], we similarly concluded that patient videos helped staff to gain a more individualized understanding of patients. A deeper understanding of patients as individuals likely, in part, underpinned the closer staff-patient engagement anecdotally reported by participating staff and patients.

Older adults with frailty often face multiple transitions between health and social care settings, owing to complex care needs stemming from multimorbidity [6,7]. An important application of the present intervention, as conceived by ward staff, lay in the potential to capture video footage which could be shown to other departments or providers to inform decision-making and support joined-up care. For example, staff spoke about the potential value in sharing footage with neurology specialists or with staff carrying out assessments for care home placements. For the purposes of this pilot study, staff could only show video recordings to health care professionals outside the direct care team who visited the pilot wards in person. Should wider implementation of video-based patient records be considered, the lack of interoperability of different EPR systems, a well-documented obstacle to delivering digital interventions at scale, warrants timely address [31,32].

The integration of new digital technologies into health care services resembles a complex challenge, with tailored strategies required to promote successful adoption and sustainability of a given intervention [33]. Our findings point to a series of barriers and facilitators to the implementation of video-based patient records, including those pertaining to staff engagement, workflow, and the adequacy of reminders and prompting used to encourage staff to capture and view patient videos. So that potential enhancements to patient care can be realized, theoretically informed strategies drawing upon behavioral science or implementation science frameworks are likely required to encourage uptake [34].

Finally, it is well documented that severely frail patients and people who are unable to consent to participation are seldom included within research studies [35,36]. Our findings reinforce existing literature which points to the need for careful ethical review of procedures for patient inclusion [37], to increase opportunities for such patient populations to contribute to applied clinical and health services research. This is so that they are not excluded from the development of innovations which may result in enhanced care.

### Strengths and Limitations

Strengths of this study lie in its representation of patient populations who are underrepresented and underserved in clinical research. This includes older adults with severe frailty and people who lack the capacity to consent to research owing to cognitive impairment [35]. Exclusion of such patients from clinical trials is widespread, thus obstructing the potential to develop improved health care services and interventions that meet the needs of these populations [36]. High quality and comprehensiveness in qualitative data collection components were indicated according to multiple dimensions of information power [24]. For instance, data were sufficiently comprehensive given our narrow study aims, high sample specificity while capturing variation in patient demographic and clinical characteristics, and owing to the application of established theory. We ensured rigor in our analysis of interview data through the Framework Analysis approach [22], applying an existing theoretically informed framework to guide data charting [23], while inductively introducing new codes into the coding scheme to characterize each construct. Further strengths were in our approach to PPIE, ensuring that lived experience perspectives guided the study throughout the research cycle.

Issues in interpreting Isla Health Digital Pathway Platform metadata, such that video view figures were estimates that cannot be determined with exactness, represent an important limitation. Other limitations pertain to the study design. The single-site, nonrandomized nature of this research and the lack of a comparator group should be taken into account when considering our findings. While we appraised the quality of interview dialogue to be high overall, we hold that dialogue flow was at times impacted within the busy ward environment in which data were gathered. Interviews with patients were often inadvertently interrupted by staff or visitors, while staff were regularly distracted by other tasks (eg, incoming telephone calls) while being interviewed. We also note that among participating patients with the capacity to consent to an optional interview during enrollment, a number of these individuals were not interviewed due to reasons such as being uncontactable within 2 weeks of leaving the hospital, deterioration in cognitive state, and lack of interpreter availability at the point of discharge. Had it been possible to interview these individuals, it is plausible that a wider range of perspectives on the intervention may have surfaced, with greater information power to permit between-case analysis. Nonetheless, we offer a robust evaluation of intervention feasibility, applying our findings stringently to decision-making about progression to a definitive trial.

### Implications

Study findings provide evidence that the use of video-based patient records within direct care delivery for older adults with frailty was largely deemed to be acceptable. We also offer compelling preliminary insights based on participating patient, carer, and clinical staff opinions as to the potential effectiveness of this intervention in the acute medical inpatient ward context. When implemented successfully, our findings suggest that the intervention could contribute to improved care quality and safety. However, the feasibility of intervention implementation must be questioned, given estimated video view metrics and anecdotal evidence from staff interviews, which indicated that uptake among staff in terms of capturing and viewing patient videos did not reach a threshold to permit full realization of benefits. Evidence for ethicality and a lack of adverse outcomes suggests that the intervention could be considered appropriate for use within other clinical services when indicated, for example, as part of efforts to empower patients and to promote person-centered care. Further professional and regulatory guidance to support clinical staff in the implementation of such an intervention is, however, warranted [16].

### Conclusions

Addressing the primary objective of this pilot study, the video-based patient record intervention was found to be largely acceptable to older inpatients with frailty, carers, and staff. Participants also held promising opinions about possible intervention impacts. Optimizing assessments, enhancing care continuity, and empowering patients were considered important potential benefits of using patient videos within direct care. No adverse events were reported. Findings about patient diversity, alongside barriers and facilitators to intervention implementation, support encouraging indications overall as to the feasibility of implementing such an intervention within this care context. However, apparent limited engagement with patient videos after their capture during the pilot phase would likely obstruct our ability to measure intervention efficacy in future research. As such, we cannot endorse progression to a definitive trial to examine the effectiveness of patient videos for improving safety and quality of care transitions. Nevertheless, our findings provide an initial indication that the intervention could be ethically implemented into routine practice, with suitable ethical safeguards in place.

## Supplementary material

10.2196/77318 Multimedia Appendix 1 Procedure for patient screening and enrollment.

10.2196/77318 Multimedia Appendix 2 Video evaluation questionnaire completed by ward staff upon viewing a patient video.

10.2196/77318 Multimedia Appendix 3 Interview guide for patients and carers.

10.2196/77318 Multimedia Appendix 4 Interview guide for ward staff.

10.2196/77318 Multimedia Appendix 5 Component constructs of the Theoretical Framework of Acceptability of Healthcare Interventions.

## Abbreviations

CONSORT: Consolidated Standards of Reporting Trials
EPR: electronic patient record
MDT: multidisciplinary team
NHS: National Health Service
NIHR: National Institute for Health and Care Research
PPIE: patient and public involvement and engagement
TIDieR: Template for Intervention Description and Replication
