# Supplementary material for: Acceptability, Feasibility, and Perceived Effectiveness of Video-Based Patient Records for Supporting Care Delivery to Older Adults With Frailty: Nonrandomized Mixed Methods Pilot Study
Source: J Med Internet Res. 2026 Jan 6;28:e77318. doi: 10.2196/77318 (PMC12774403; doi:10.2196/77318)

**Multimedia Appendix 1.** Procedure for patient screening and enrollment

This is a Multimedia Appendix to a full manuscript published in the J Med Internet Res. For full copyright and citation information see <https://doi.org/10.2196/77318>.


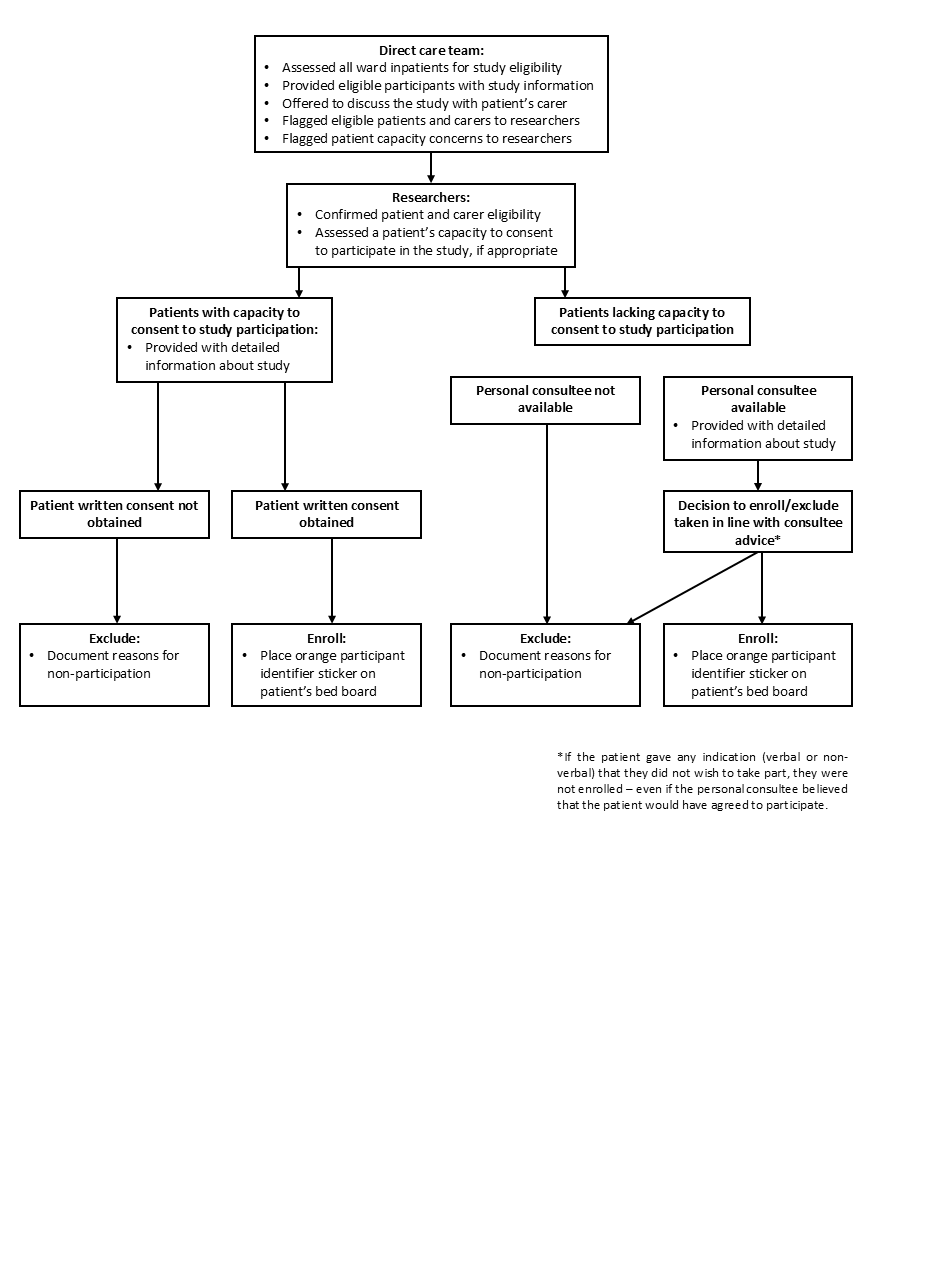

Supplement: Multimedia Appendix 1 [file jmir-v28-e77318-s001.docx]
