# Supplementary material for: Acceptability, Feasibility, and Perceived Effectiveness of Video-Based Patient Records for Supporting Care Delivery to Older Adults With Frailty: Nonrandomized Mixed Methods Pilot Study
Source: J Med Internet Res. 2026 Jan 6;28:e77318. doi: 10.2196/77318 (PMC12774403; doi:10.2196/77318)
Supplement: Multimedia Appendix 2 [file jmir-v28-e77318-s002.docx]

**Multimedia Appendix 2.** Video evaluation questionnaire completed by ward staff upon viewing a patient video

This is a Multimedia Appendix to a full manuscript published in the J Med Internet Res. For full copyright and citation information see <https://doi.org/10.2196/77318>.

**Video Evaluation Questionnaire**

**1. Which aspect(s) of the patient’s condition/functional ability are captured?**

Mobility

Transfers

Eating and drinking

Alertness

Cognitive function

Behaviour

Communication

Clinical examination

Patient preferences

Other: please specify……….

**2. Is video quality suitable for clinical interpretation?**

Yes

No

To some extent

Please explain your answer <free-text comment>

**3. Comment on the video length:**

Too long

Too short

Just right

Please explain your answer <free-text comment>

**4. How useful is this video for supporting patient assessment?**

Very useful

Somewhat useful

Useless

Please explain your answer <free-text comment>

**5. How useful is this video for supporting decision-making?**

Very useful

Somewhat useful

Useless

Please explain your answer <free-text comment>

**6. How useful is this video for communicating patient information to colleagues?**

Very useful

Somewhat useful

Useless

Please explain your answer <free-text comment>

**7. Do you want to see more patient videos in future?**

Yes

No

Please explain your answer <free-text comment>
