# Supplementary material for: Acceptability, Feasibility, and Perceived Effectiveness of Video-Based Patient Records for Supporting Care Delivery to Older Adults With Frailty: Nonrandomized Mixed Methods Pilot Study
Source: J Med Internet Res. 2026 Jan 6;28:e77318. doi: 10.2196/77318 (PMC12774403; doi:10.2196/77318)
Supplement: Multimedia Appendix 3 [file jmir-v28-e77318-s003.docx]

**Multimedia Appendix 3.** Interview guide for patients and carers

This is a Multimedia Appendix to a full manuscript published in the J Med Internet Res. For full copyright and citation information see <https://doi.org/10.2196/77318>.

**Interview Topic Guide: Patients & Carers/Consultees**

*Affective Attitude*

How did you feel about the idea of video-based patient records before taking part in this study? And how do you feel about video-based patient records now?

*Burden*

How much effort did it take for you to participate in the video-recordings?

*Ethicality*

To what extent do you think it is fair for patients like you to be video recorded by healthcare professionals? To what extent do you think there are moral or ethical consequences?

*Perceived effectiveness*

What impacts of video-based patient records have you noticed? To what extent do you think being video recorded has improved your care?

[Prompt. For example - in terms of:

- The accuracy of your assessments
- Communicating information about you to your care team
- How joined up your care is
- How personalised your care feels]

*Intervention coherence*

To what extent does being video recorded by your care team make sense to you? Is it clear to you how your videos might support your assessment and care?

*Self-efficacy*

How confident do you feel about participating in video recordings?

*Process Evaluation*

What worked well and what didn’t work so well with regards to patient videos? What are the barriers and what facilitates the video-recording process?

*Future application*

In what circumstances do you think video-based patient records could be used in the future?

*General Acceptability*

How acceptable are video-based patient records to you:

Completely unacceptable

Unacceptable

No opinion

Acceptable

Completely acceptable

What are your overriding thoughts about video-based patient records?
