# Supplementary material for: Acceptability, Feasibility, and Perceived Effectiveness of Video-Based Patient Records for Supporting Care Delivery to Older Adults With Frailty: Nonrandomized Mixed Methods Pilot Study
Source: J Med Internet Res. 2026 Jan 6;28:e77318. doi: 10.2196/77318 (PMC12774403; doi:10.2196/77318)
Supplement: Multimedia Appendix 4 [file jmir-v28-e77318-s004.docx]

**Multimedia Appendix 4.** Interview guide for ward staff

This is a Multimedia Appendix to a full manuscript published in the J Med Internet Res. For full copyright and citation information see <https://doi.org/10.2196/77318>.

**Interview Topic Guide: Ward Staff**

*Affective Attitude*

How did you feel about the idea of video-based patient records before taking part in this pilot? And how do you feel about video-based patient records now?

*Burden*

How much time and effort were needed for you to record and view patients’ video records?

*Ethicality*

To what extent do you think it is fair for patients with frailty to be video recorded by healthcare professionals? To what extent do you think there are moral or ethical consequences?

*Perceived effectiveness*

What impacts of video-based patient records have you noticed? To what extent do you think video-based patient records support you to assess and care for your patients?

*[Prompts:*

- *Patient assessment & clinical decision-making*
- *Communication with other team members about patients*
- *Continuity of care*
- *Person-centred care]*

*Intervention coherence*

To what extent does implementing video-based records make sense to you? Is it clear to you how video-based patient records might support assessment and care delivery?

*Self-efficacy*

How confident do you feel about capturing video-recordings of patients and using the videos to support patient assessment and care?

*Process Evaluation*

What worked well and what didn’t work so well with regards to patient videos? What are the barriers and what facilitates the video-recording process?

*Future application*

In what circumstances or clinical settings do you think video-based patient records could be used in the future?

*General Acceptability*

How acceptable are video-based patient records to you:

Completely unacceptable

Unacceptable

No opinion

Acceptable

Completely acceptable

What are your overriding thoughts about video-based patient records?
