# Supplementary material for: Acceptability, Feasibility, and Perceived Effectiveness of Video-Based Patient Records for Supporting Care Delivery to Older Adults With Frailty: Nonrandomized Mixed Methods Pilot Study
Source: J Med Internet Res. 2026 Jan 6;28:e77318. doi: 10.2196/77318 (PMC12774403; doi:10.2196/77318)
Supplement: Multimedia Appendix 5 [file jmir-v28-e77318-s005.docx]

**Multimedia Appendix 5.** Component constructs of the Theoretical Framework of Acceptability of Healthcare Interventions

This is a Multimedia Appendix to a full manuscript published in the J Med Internet Res. For full copyright and citation information see <https://doi.org/10.2196/77318>.

Sekhon’s Theoretical Framework of Acceptability of Healthcare Interventions

| **Construct** | **Definition** |
| --- | --- |
| **Affective attitude** | How an individual feels about the intervention |
| **Burden** | The amount of effort required to participate in the intervention |
| **Ethicality** | The extent to which the intervention has a good fit with an individual’s value system |
| **Intervention conference** | The extent to which the participant understands the intervention and how it works |
| **Opportunity costs** | The extent to which benefits, profits, or values must be given up to engage in the intervention |
| **Perceived effectiveness** | The extent to which the intervention is perceived to achieve its purpose |
| **Self-efficacy** | The participant’s confidence that they can perform the behaviors required to participate in the intervention |
|  |  |

Sekhon M, Cartwright M, Francis JJ. Acceptability of healthcare interventions: an overview of reviews and development of a theoretical framework. *BMC Health Serv Res*. 2017;17(1):1-13. doi:10.1186/s12913-017-2031-8
